# Understanding the biological mechanisms of cancer treatment-induced cardiac toxicity

**DOI:** 10.1016/j.ahjo.2022.100177

**Published:** 2022-07-13

**Authors:** Eric H. Yang, Rachel E. Ohman

**Affiliations:** UCLA Cardio-Oncology Program, Division of Cardiology, Department of Medicine, University of California at Los Angeles, Los Angeles, CA, United States of America

**Keywords:** Cardio-oncology, Cardiotoxicity, Healthcare disparities

## Abstract

While many strides have been made in the multidisciplinary science of Cardio-Oncology, gaps in knowledge remain despite these advances to identify optimal strategies of detection and treatment of cancer treatment-associated cardiotoxicity. Many opportunities are available for advocates from all avenues of the field to transform cardio-oncology from a reactionary to a preventative science.

As the multidisciplinary field of cardio-oncology continues to gain momentum, there has been a rapid growth in programs providing care to a growing cancer population in the United States and beyond [1]. Initiatives focused on understanding the biological mechanisms and underpinnings of cancer biology and treatment-associated cardiovascular toxicity in the clinical, basic science, and translational arenas of our research efforts are critical [2]. While there continue to be impressive gains made in our mechanistic understanding of cardiotoxicity in both historical (i.e., anthracyclines, anti-HER2, radiation treatments) and modern (tyrosine kinase inhibitors, immunotherapy) therapies, there remain gaps in understanding in 1) risk characterization in identifying vulnerable populations and 2) determining effective cardioprotective strategies undergoing cancer treatments (Table 1). What compounds this challenge is the unique combination of cardiovascular risk factors and elements of cancer biology and the overwhelming spectrum of treatment regimens in determining optimal strategies in detecting and treating cardiotoxicity (Fig. 1).

**Table 1:** Some cardiotoxic states and gaps in understanding by agents and disease states.

Cardiotoxic disease states	Therapeutic agents	Gaps in understanding by disease state
Heart failure or left ventricular dysfunction	*Anthracyclines*	Impact of disparities, preexisting CVD risk factors and treatments, and gaps in care on CTRCD risk
	*Monoclonal antibodies*	
	*Alkylating agents*	Identification of cancer patients most at risk for CTRCD
	*Tyrosine kinase inhibitors*	
	*Thoracic radiation*	Minimal efficacy of neurohormonal cardioprotective strategies in reducing CTRCD
	*Proteasome inhibitors*	
	*CART-cell therapy*	Potential cardioprotective effects of more novel heart failure treatments (i.e., ARNI, SGLT2 inhibitors)
	*Microtubule inhibitors*	
	*Immune checkpoint inhibitors*	
Hypertension	*Tyrosine kinase inhibitors (vascular endothelial growth factor inhibitors [VEGFi])*	Impact of disparities, preexisting CVD risk factors and treatments, and gaps in care on VEGFi-induced hypertension
	*Monoclonal antibodies*	Absence of randomized control trials testing optimal antihypertensive agents for treatment of VEGFi-induced hypertension
		Long-term ASCVD/heart failure risk
Atherosclerotic vascular disease (ASCVD)	*Tyrosine kinase inhibitors*	Impact of disparities, preexisting CVD risk factors and treatments, and gaps in care on cancer treatment-associated ASCVD
	*Antimetabolites*	Suspected inaccuracy of traditional risk factor models and calculators (i.e., Pooled cohort equations, Multiethnic Study of Atherosclerosis) in cancer populations
	*Alkylating agents*	Optimal imaging and treatment strategies (i.e., statins, antiplatelet treatments) for ASCVD in cancer populations
	*Monoclonal antibodies*	Variability of ASCVD risk depending on preexisting risk factor profile, cancer type, and treatment agents
	*Immune checkpoint inhibitors*	
	*Thoracic radiation*	Long-term ASCVD risk of changing radiation techniques
Arrhythmias	*CART-cell therapy*	Mechanisms of arrhythmias precipitated by cancer treatments
	*Thoracic radiation*	
	*Anthracyclines*	
	*Antimetabolites*	
	*Microtubule inhibitors*	Suboptimal anticoagulation strategies due to drug-drug interactions with both warfarin and direct-acting oral anticoagulant agents
	*Alkylating agents*	
	*Monoclonal antibodies*	
	*Tyrosine kinase inhibitors (*i.e. *ibrutinib)*	Long-term arrhythmia and stroke risk (i.e., atrial fibrillation) of cancer treatments
	*Proteasome inhibitors*	
Myocarditis	*Immune checkpoint inhibitors (ICI)*	Elucidation of risk factors and identification of patient populations at risk for ICI-associated myocarditis
		Optimal strategies (e.g., imaging, biomarker) for the detection and diagnosis of ICI-associated myocarditis
		Absence of randomized control trials in evaluating treatment strategies for ICI myocarditis Decipher toxicities associated with other immunotherapies

ARNI = angiotensin receptor neprilysin inhibitor, ASCVD = atherosclerotic cardiovascular disease, CAR = chimeric antigent receptor, CVD = cardiovascular disease, CTRCD = cancer treatment-associated cardiac dysfunction, ICI = immune checkpoint inhibitor, SGLT2 = sodium glucose transport protein 2.

**Fig. 1:**
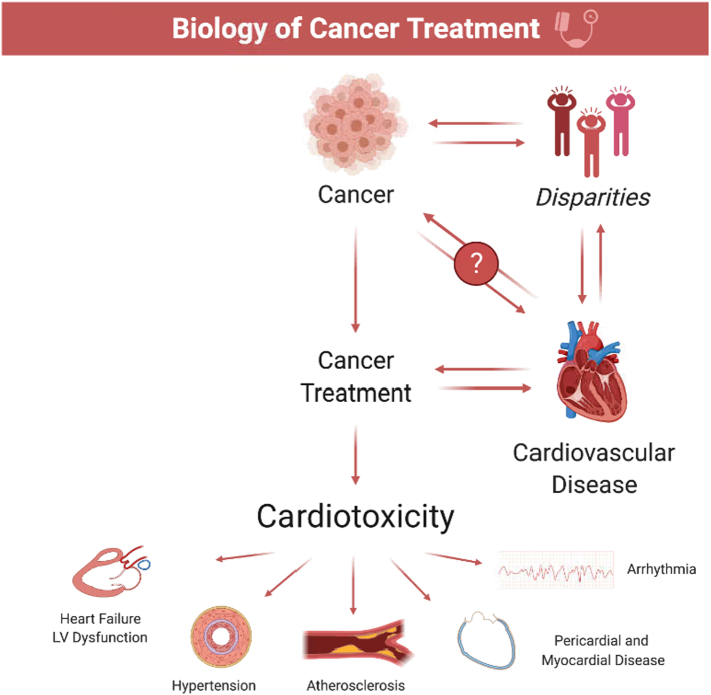
The biology of cancer treatments. The unique combination of cardiovascular risk factors and elements of cancer biology and overwhelming spectrum of treatment regimens that optimal strategies for detecting and treating cardiotoxicity.

For instance, more insights have been made in the role of anthracyclines—regarded as an essential agent in the treatment of hematologic malignancies, sarcomas, and breast cancer—an excessive generation of reactive oxygen species, detrimental effects by binding to topoisomerase 2β and subsequent impact on cell survival and metabolic pathways [3]; thus leading to cardiac dysfunction and heart failure (HF). In addition, the contribution of anti-HER2 treatments (i.e., trastuzumab) also disrupt mechanisms of cardiac homeostasis and may lead to decreased cardiomyocyte proliferation and survival. Neurohormonal therapies, which have shown overall efficacy in the population with heart failure with reduced ejection fraction, have the potential to counteract these proapoptotic pathways and negative remodeling. However, when studying these cardioprotective therapies in randomized controlled trials, the totality of these trials do not seem to yield an impressive benefit in reducing the risk of left ventricular ejection fraction (LVEF) decline [4], [5]. Further avenues of investigation are warranted to identify which 1) mechanisms of cardiotoxicity, 2) cancer population, and 3) risk factors will benefit from such therapies.

Another drug class has led to significant improvements in survival in the gastrointestinal/genitourinary cancer population with the use of vascular endothelial growth factor inhibitors (VEGFi), a type of tyrosine kinase inhibitor. While their anticancer effects focus on inhibiting tumor angiogenesis which is mediated by VEGF, undesired effects can occur from anti-VEGF activity with other growth factors and receptors, including platelet-derived growth factor, fibroblast growth factor, c-Kit, and Flt-3 [6].

The most clinically evident toxicity exhibited by VEGFi is hypertension; while mechanisms surrounding this phenomenon are unclear, proposed mechanisms stem from the role of VEGF as a regulator of vascular tone and blood pressure. Imbalances between vasoconstrictor (endothelin-1) and vasodilator (nitric oxide), oxidative stress, renal toxicity, microvascular rarefaction, are thought to be potential causes of VEGFi induced hypertension [6], [7]. While the optimal antihypertensive strategy has not been determined yet, a retrospective institutional study suggested an association of more optimal blood pressure control with calcium channel blockers and potassium-sparing diuretic agents [7]. Further prospective trials are needed to investigate the best treatment strategies to attenuate this known toxicity which is critical to continuing cancer treatments and potentially improving our understanding of the traditional causes of hypertension.

Immunotherapy has also skyrocketed as a heralded treatment that has offered hope in malignancies with historically poor prognoses, particularly lung cancer and melanoma. Immune checkpoint inhibitors (ICI) are immunotherapies that function by releasing the “brakes” that tumor cells can typically use to suppress T-cell activation via co-stimulation by the cytotoxic T lymphocyte antigen 4 (CTLA-4) or triggering the programmed death 1 (PD-1) receptor. However, this activation cascade and cross-reaction of antitumor T-cells with other organs can lead to immune-related adverse events (IRAEs), with potential involvement of the cardiovascular system [8], [9]. While the more dramatic and rare manifestation of ICI-associated myocarditis has generated much attention, there are emerging signals of increased cardiovascular events in patients on ICI therapy, including myocardial infarction (MI), stroke, and need for coronary revascularization [10]. Indeed, there is an increased interest in evaluating the role of the immune system and inflammation, particularly with immunotherapy, on atherosclerosis metabolism. While this therapy is important for selected cancer patients, it is key to understanding its long-term effects on other traditional forms of cardiovascular disease in order to investigate interventional therapies that can attenuate short- and long-term cardiovascular event rates.

There is also emerging evidence about elevated cancer risk in patients with cardiovascular disease [11], or the theme of “reverse Cardio-Oncology.” Basic science studies have shown potential links to heart failure-induced circulating factors and immune cell reprogramming after MI that may promote or accelerate tumor growth [12]. In addition, insights into clonal hematopoiesis of indeterminate potential (CHIP), a known somatic mutation in the cancer community, continue to show links as a possible precursor to CVD, where older adults without cancer with CHIP mutations have a 40 % higher risk of CVD [13], [14], and up to a 25 % higher risk of heart failure compared to non-CHIP controls [15]. CHIP may represent a nontraditional risk factor for cardiovascular and cancer risk and warrants further study.

In conclusion, while many exciting strides have been made in the multidisciplinary science of Cardio-Oncology, more mechanistic questions have arisen, and gaps in knowledge remain despite these advances to identify optimal strategies of detection and treatment of cancer treatment-associated cardiotoxicity. Many opportunities are available for advocates from all avenues of the field to transform cardio-oncology from a reactionary to a preventative science.

## Declaration of competing interest

The authors declare that they have no known competing financial interests or personal relationships that could have appeared to influence the work reported in this paper.
